# Redox regulation of the Calvin–Benson cycle: something old, something new

**DOI:** 10.3389/fpls.2013.00470

**Published:** 2013-11-25

**Authors:** Laure Michelet, Mirko Zaffagnini, Samuel Morisse, Francesca Sparla, María Esther Pérez-Pérez, Francesco Francia, Antoine Danon, Christophe H. Marchand, Simona Fermani, Paolo Trost, Stéphane D. Lemaire

**Affiliations:** ^1^Laboratoire de Biologie Moléculaire et Cellulaire des Eucaryotes, FRE3354 Centre National de la Recherche Scientifique, Institut de Biologie Physico-Chimique, Université Pierre et Marie CurieParis, France; ^2^Laboratory of Plant Redox Biology, Department of Pharmacy and Biotechnology (FaBiT), University of BolognaBologna, Italy; ^3^Department of Chemistry “G. Ciamician”, University of BolognaBologna, Italy

**Keywords:** Calvin–Benson cycle, CO_2_ fixation, thioredoxin, glutaredoxin, glutathionylation, nitrosylation, photosynthesis, redox regulation

## Abstract

Reversible redox post-translational modifications such as oxido-reduction of disulfide bonds, S-nitrosylation, and S-glutathionylation, play a prominent role in the regulation of cell metabolism and signaling in all organisms. These modifications are mainly controlled by members of the thioredoxin and glutaredoxin families. Early studies in photosynthetic organisms have identified the Calvin–Benson cycle, the photosynthetic pathway responsible for carbon assimilation, as a redox regulated process. Indeed, 4 out of 11 enzymes of the cycle were shown to have a low activity in the dark and to be activated in the light through thioredoxin-dependent reduction of regulatory disulfide bonds. The underlying molecular mechanisms were extensively studied at the biochemical and structural level. Unexpectedly, recent biochemical and proteomic studies have suggested that all enzymes of the cycle and several associated regulatory proteins may undergo redox regulation through multiple redox post-translational modifications including glutathionylation and nitrosylation. The aim of this review is to detail the well-established mechanisms of redox regulation of Calvin–Benson cycle enzymes as well as the most recent reports indicating that this pathway is tightly controlled by multiple interconnected redox post-translational modifications. This redox control is likely allowing fine tuning of the Calvin–Benson cycle required for adaptation to varying environmental conditions, especially during responses to biotic and abiotic stresses.

## Introduction

Redox post-translational modifications (PTM) of cysteine residues play a prominent role in the regulation of cell metabolism and signaling in all organisms. Indeed, cysteine residues can undergo different states of oxidation such as sulfenic (−SOH), sulfinic (−SO_2_H) and sulfonic acids (−SO_3_H) but also protein disulfide bonds (intra- or intermolecular, −SS−), S-thiolation (mainly glutathionylation, −SSG) or nitrosylation (−SNO). Most of these modifications are controlled by small disulfide oxidoreductases named thioredoxins (TRXs) and glutaredoxins (GRXs).

The importance of redox PTMs has been recognized very early in plants through studies aimed at understanding the mechanisms underlying the regulation of enzymes of the Calvin–Benson cycle (CBC). This pathway is responsible for CO_2_ fixation by photosynthetic organisms and is therefore at the basis of our food chain. The CBC, or more generally photosynthesis, fuels all life on Earth with energy (Pfannschmidt and Yang, 2012). Without photosynthesis, no complex ecosystems and higher life forms including man would exist (Blankenship, 2002; Buchanan et al., 2002a).

**Figure 1:**
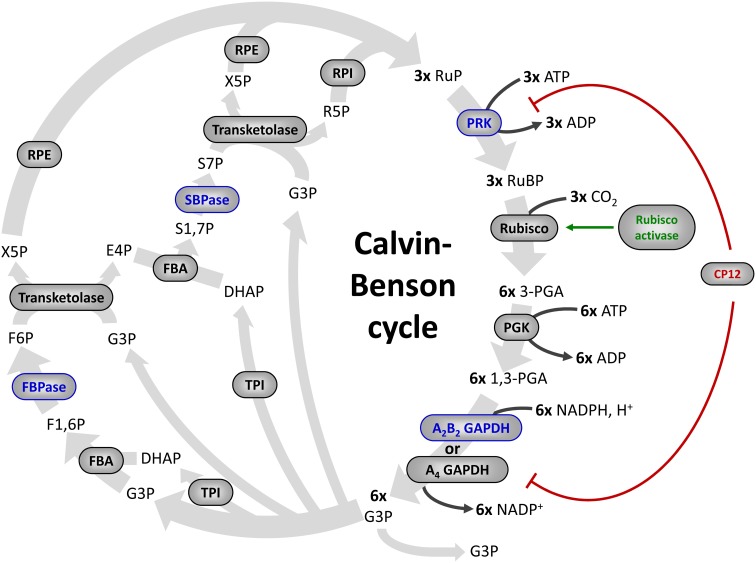
**The Calvin–Benson cycle.** The eleven enzymes of the Calvin–Benson cycle are indicated in gray ellipses. Four enzymes (in blue) are activated directly by TRXs. Some proteins that control the activity of Calvin–Benson cycle enzymes are also regulated by TRXs: Rubisco activase (in green), and CP12 (in red), which forms a complex with PRK and A_4_-GAPDH and inhibits both enzymes. Enzymes: Rubisco, ribulose-1,5-bisphosphate Carboxylase/Oxygenase; PGK, phosphoglycerate kinase; GAPDH, glyceraldehyde-3-phosphate dehydrogenase; TPI, triose phosphate isomerase; FBA, fructose-1,6-bisphosphate aldolase; FBPase, fructose-1,6-bisphosphatase; TK, transketolase; SBPase, sedoheptulose-1,7-bisphosphatase; RPE, ribulose-5-phosphate 3-epimerase; RPI, ribose-5-phosphate isomerase; PRK, phosphoribulokinase. Metabolites, RuBP, ribulose-1,5-bisphosphate; 3-PGA, 3-phosphoglycerate; 1,3-PGA, 1,3-bisphosphoglycerate; G3P, glyceraldehyde-3-phosphate; DHAP, dihydroxyacetone phosphate; F1,6P, fructose-1,6-bisphosphate; F6P, fructose-6-phosphate; X5P, xylulose-5-phosphate; E4P, erythrose-4-phosphate; S1,7P, sedoheptulose-1,7-bisphosphate; S7P, sedoheptulose-7-phosphate; R5P, ribulose-5-phosphate; RuP, ribulose-5-phosphate.

After the initial discovery of the pathway by Bassham et al. (1950), the enzymes of the cycle were purified and characterized from diverse sources including C3 and C4 plants, algae and cyanobacteria (Figure 1). In the 60s, the activity of several enzymes of the CBC was found to be regulated by light. These enzymes were found to have a low activity in the dark and to be activated in the light. Investigation of the molecular mechanism of this light-dependent regulation led to the identification of the ferredoxin/thioredoxin (Fd/TRX) system that plays a crucial role in numerous redox- and light-dependent reactions in chloroplasts. Four enzymes of the CBC regulated by light were shown to contain a regulatory disulfide oxidized in the dark and reduced in the light by TRX. This reduction allows transition from a low active form to a fully active enzyme. Additional proteins were also recognized as TRX-regulated targets such as proteins involved indirectly in the regulation of the CBC, in light-dependent ATP production or in diverse carbon metabolism pathways (Lemaire et al., 2007; Schürmann and Buchanan, 2008). All these enzymes are regulated by light through TRX-dependent reduction of disulfide bonds. The mechanisms of this light-dependent regulation of carbon assimilation enzymes are considered as the best characterized mechanisms of redox signaling in photosynthetic organisms (Foyer and Noctor, 2005) since they were investigated in detail at the molecular and structural level in different model systems.

However, recent proteomic studies are now challenging this rather simple view. Indeed, proteomic analyses aimed at identifying new TRX targets suggested that not only four but all CBC enzymes might be redox-regulated through mechanisms likely involving cysteine residues (Lemaire et al., 2007; Lindahl et al., 2011). Moreover, all enzymes of the cycle were also identified by proteomic approaches as potential targets of nitrosylation and glutathionylation, two redox PTMs whose importance in signaling and regulation has emerged recently (Zaffagnini et al., 2012a). This suggests that the CBC is likely regulated by a complex network of interconnected redox PTMs that remain to be characterized. In this review we will provide an overview of our current knowledge on the redox regulation of CBC enzymes and related proteins and discuss the potential importance of novel types of redox modifications on our understanding of the regulation of CO_2_ fixation in photosynthetic organisms.

## The ferredoxin/thioredoxin system

**Figure 2:**
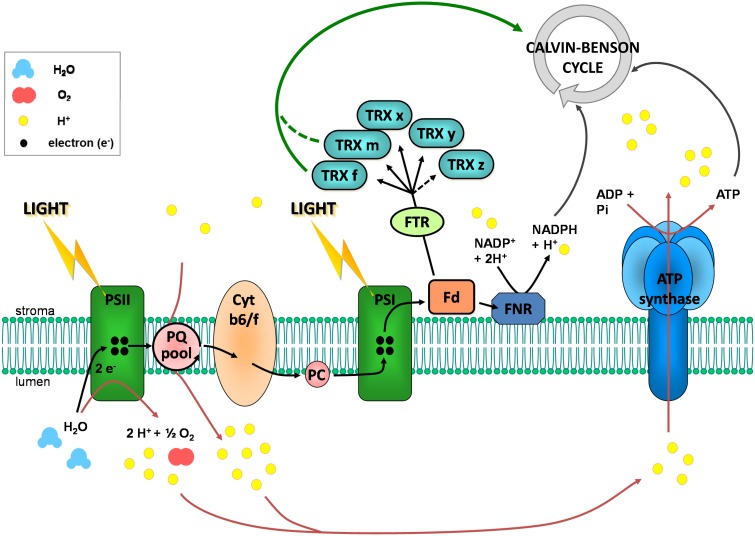
**The photosynthetic electron transfer chain and the reduction of chloroplastic TRXs.** In the thylakoid membrane, when photosystem II (PSII) is excited by absorption of a photon light energy, the reaction center chlorophyll molecule transiently loses an electron. This electron is transmitted to the plastoquinone pool (PQ) which takes a proton from the stroma. Upon oxidation, the reaction center chlorophyll is a very strong oxidizing agent which is able to accept electrons from water, resulting in oxygen and protons production in the lumen. The chlorophyll can then be excited again. Reduced plastoquinone can move through the membrane from PSII to cytochrome b6/f (Cyt b6/f). There, plastoquinone is oxidized and its proton is released in the lumen, leading to a proton transport from stroma to lumen. Its electron is further transferred to photosystem I (PSI) via cytochrome b6/f and plastocyanin (PC). This electron transfer allows reduction of excited PSI reaction center chlorophyll. Upon excitation, this chlorophyll gives its electron to stromal ferredoxin (Fd) which can reduce chloroplastic thioredoxins (TRX) via the ferredoxin-thioredoxin reductase (FTR) and NADP^+^ via the ferredoxin NADP reductase (FNR). Water photolysis and proton transport via plastoquinones contribute to the establishment of a proton gradient between stroma and lumen. This gradient is used as an energy source by the ATP synthase for ATP synthesis.

**Figure 3:**
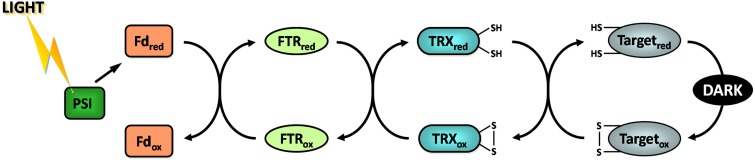
**The ferredoxin/thioredoxin system.** Fd, ferredoxin; FTR, ferredoxin thioredoxin reductase; PSI, photosystem I; ox, oxidized; red, reduced.

During the 60s and 70s numerous studies have reported that the activity of four CBC enzymes was regulated by light (reviewed in Buchanan, 1980, 1991; Schürmann and Jacquot, 2000; Lemaire et al., 2007; Schürmann and Buchanan, 2008; Buchanan et al., 2012). These enzymes were phosphoribulokinase (PRK), glyceraldehyde-3-phosphate dehydrogenase (GAPDH), fructose-1,6-bisphosphatase (FBPase), and sedoheptulose-1,7-bisphosphatase (SBPase) (Figure 1). These four enzymes were found to have a low activity in the dark and to be activated under illumination. During the same period, a similar regulation was reported for NADP-malate dehydrogenase (NADP-MDH) (Johnson and Hatch, 1970) and chloroplast ATP synthase (McKinney et al., 1978, 1979). NADP-MDH is involved in CO_2_ fixation in C4 plants and in the export of excess reducing power from the chloroplast in C3 plants while chloroplast ATP synthase produces ATP equivalents required for CO_2_ fixation by the CBC. The analysis of the molecular mechanisms underlying these light-dependent activations were mostly performed in the laboratory of Prof. Bob Buchanan in Berkeley and led to the identification of the so-called ferredoxin/thioredoxin system (Buchanan, 1991; Buchanan et al., 2002b). This system is composed of three types of chloroplast soluble proteins located in the stroma: ferredoxin (Fd), ferredoxin/thioredoxin reductase (FTR) and thioredoxin (TRX) (Figure 2). Upon illumination, ferredoxin is reduced by the photosynthetic electron transfer chain at the level of photosystem I (PSI). Chloroplastic Fd is located at a metabolic crossroad in the chloroplast and once reduced can transfer its electron(s) to enzymes involved in diverse metabolic pathways including photoreduction of NADP through ferredoxin/NADP reductase (FNR), reduction of sulfites and nitrites, lipid biosynthesis, hydrogen production (Winkler et al., 2010) and photosynthetic cyclic electron flow via ferredoxin-plastoquinone reductase (Hertle et al., 2013). Several isoforms of PSI-reduced Fd are present in photosynthetic organisms: 4 distinct Fd were described in the land plant *Arabidopsis thaliana* whereas 6 isoforms were reported in the green alga *Chlamydomonas reinhardtii*. These Fds are not equivalent as they exhibit some specificities toward their target enzymes and distinct expression profiles (Hanke et al., 2004; Terauchi et al., 2009). Under optimal growth conditions, most of the electron flux is likely directed to FNR to produce, in the form of NADPH, the reducing power required for CO_2_ fixation by the CBC. Part of the reduced Fd pool also transfers electrons to FTR which can subsequently reduce the disulfide bond present in the active site of several types of chloroplastic TRXs. FTR is a thin and flat [4Fe-4S] enzyme interacting with Fd on one side and TRX on the other side (Dai et al., 2007). A first Fd molecule binds FTR and transfers one electron to the FTR [4Fe-4S] cluster. An intermediate is then formed in which one sulfur atom of FTR active site is free to attack the disulfide of TRX and the other sulfur atom forms a fifth ligand for an iron atom in the FTR [4Fe-4S] center. A second Fd molecule then delivers a second electron that cleaves the FTR-TRX mixed-disulfide. FTR is therefore unique in its use of a [4Fe-4S] cluster and a proximal disulfide bridge in the conversion of a light signal into a thiol signal (Dai et al., 2007). Once reduced, TRXs are able to reduce regulatory disulfides on their target enzymes, including PRK, GAPDH, FBPase, and SBPase, allowing their activation upon illumination through reduction of their regulatory disulfide by the Fd/TRX system (Figure 3).

Originally identified for their ability to activate enzymes of the CBC, TRXs were later found to contribute to the regulation of diverse chloroplastic enzymes involved in other metabolic pathways such as ATP synthase which produces ATP in the light, Acetyl-CoA carboxylase which catalyzes the first committed step of fatty acid biosynthesis, ADP-glucose pyrophosphorylase, glucan:water dikinase and beta-amylase BAM1 involved in starch metabolism (Ballicora et al., 2000; Geigenberger et al., 2005; Mikkelsen et al., 2005; Sparla et al., 2006) or the oxidative pentose phosphate pathway enzyme glucose-6-phosphate dehydrogenase (Wenderoth et al., 1997; Nee et al., 2009). In addition to their role in the control of metabolic enzymes through reduction of regulatory disulfides, TRXs also play a major role in the detoxification of reactive oxygen species (ROS) and the maintenance of redox homeostasis in the chloroplast. Indeed, TRXs serve as substrate and provide electrons for the regeneration of different types of antioxidant chloroplast enzymes including peroxiredoxins (PRXs) (Dietz, 2011), glutathione peroxidases (GPXs) (Navrot et al., 2006) and methionine sulfoxide reductases (Tarrago et al., 2009). Early studies on the regulation of FBPase and NADP-MDH led to the identification of two types of TRX named TRXf and TRXm due to their substrate specificity (Jacquot et al., 1978; Wolosiuk et al., 1979). Indeed, TRXf was initially found to activate FBPase while TRXm appeared to preferentially activate NADP-MDH. TRXf was also found to be more effective than TRXm for the reduction of all other CBC enzymes (Wolosiuk et al., 1979).

The availability of genome sequences revealed the existence of three other types of TRXs in the chloroplast. TRXx was identified in Arabidopsis (Mestres-Ortega and Meyer, 1999), TRXy in Chlamydomonas (Lemaire et al., 2003), and TRXz in tomato and Arabidopsis (Rivas et al., 2004; Arsova et al., 2010; Meng et al., 2010; Schroter et al., 2010). In cyanobacteria, such as *Synechocystis* sp. PCC6803, four TRXs are present: 1 TRXm, 1 TRXx, 1 TRXy, and 1 TRX encoded by the *trxC* gene that has no ortholog in eukaryotes (Chibani et al., 2009; Pérez-Pérez et al., 2009). All five chloroplast TRX types (f, m, x, y, z) are conserved in land plants which usually contain several isoforms of each TRX type, such as Arabidopsis (2 TRXf, 4 TRXm, 1 TRXx, 2 TRXy, 1 TRXz) or poplar (1 TRXf, 8 TRXm, 1 TRXx, 2 TRXy, 1 TRXz) (Lemaire et al., 2007; Chibani et al., 2009). This multiplicity is more limited in unicellular eukaryotes such as the green algae *Chlamydomonas reinhardtii* (2 TRXf, 1 TRXm, 1 TRXx, 1 TRXy, 1 TRXz) or *Ostreococcus lucimarinus* (2 TRXf, 1 TRXm, 1 TRXx, no TRXy, 1 TRXz). Although TRXx and TRXy appear to be reduced by FTR (Bohrer et al., 2012), their biochemical properties do not allow them to activate CBC enzymes. These TRX types appear to be more specifically involved in the response to oxidative stress and the maintenance of ROS homeostasis. Indeed, TRXx and TRXy were found to be the most efficient TRXs for the reduction of PRXs, GPXs and MSRs (Collin et al., 2003, 2004; Chibani et al., 2011). TRXz has been recently characterized as a subunit of the plastid encoded RNA polymerase and plays an important role in chloroplast transcription and chloroplast development (Arsova et al., 2010; Schroter et al., 2010). In addition to this role, TRXz was suggested to be redox active although its mode of reduction remains controversial since TRXz was reported to be reduced by FTR in poplar (Chibani et al., 2011) or by other TRX types in Arabidopsis (Bohrer et al., 2012). Nonetheless, as TRXx and TRXy, TRXz appears to reduce some antioxidant enzymes but is most probably not able to reduce classical carbon metabolism targets including CBC enzymes, as demonstrated for NADP-MDH (Chibani et al., 2011; Bohrer et al., 2012). By contrast, TRXf and TRXm are clearly dedicated to the regulation of the CBC although TRXf appears to play a more prominent role. Indeed, all TRX-dependent enzymes of the CBC analyzed are exclusively or preferentially reduced by f-type TRXs. TRXm may thus play a more important role in the regulation of other chloroplastic processes and pathways such as transfer of reducing equivalents from the stroma to the thylakoid lumen (Motohashi and Hisabori, 2006, 2010) or regulation of chloroplastic proteins involved in electron transfer pathways (Courteille et al., 2013). Alternatively, TRXm isoforms may serve as alternative regulators of some CBC enzymes when TRXf activity is limiting, e.g., under oxidative stress conditions leading to protein glutathionylation in chloroplasts. Indeed, TRXf itself can be glutathionylated and consequently lose capability to regulate its targets (Michelet et al., 2005). In addition to canonical chloroplastic TRXs, numerous non chloroplastic TRX types and TRX-like isoforms are present in the genomes of photosynthetic eukaryotes (Chibani et al., 2009). Forty-one isoforms of TRXs and TRX-like proteins were reported in Arabidopsis and forty-five in poplar. Some of these TRX-related proteins are likely located in chloroplasts as shown for the peculiar CDSP32 protein composed of two TRX domains (Broin et al., 2002) and which participates in responses to oxidative stress (Rey et al., 2005; Tarrago et al., 2010).

## Molecular mechanisms of light-dependent regulation of the Calvin–Benson cycle

After the discovery of the Fd/TRX system, numerous efforts have been put on the analysis at the biochemical and structural level of the molecular mechanism underlying TRX-dependent activation of chloroplast enzymes. The insights obtained from these studies are detailed in this section.

### Calvin–Benson cycle enzymes

#### Glyceraldehyde-3-phosphate dehydrogenase (GAPDH)

GAPDH catalyzes the reversible interconversion of 1,3-bisphosphoglycerate (BPGA) into glyceraldehyde-3-phosphate (G3P) (Figure 1). At difference with NAD(H)-specific glycolytic GAPDH (GAPC) (Zaffagnini et al., 2013a,b, this series), photosynthetic GAPDH uses both NAD(H) and NADP(H) as coenzymes (Melandri et al., 1968). GAPDH was the first CBC enzyme reported to be activated by light: in leaves and chloroplast extracts subjected to short periods of illumination, the NADP(H)-dependent activity of GAPDH was found several-fold higher than in samples maintained in the dark, but the NAD(H)-GAPDH activity remained low and stable (Ziegler and Ziegler, 1965). GAPDH activation *in vivo* was strictly dependent on photosynthetic electron transport, i.e., was inhibited by the PSII inhibitor DCMU (3-(3,4-dichlorophenyl)-1,1-dimethylurea), and could be mimicked *in vitro* by addition of NADPH and the strong chemical reductant dithiothreitol (DTT) (Ziegler and Ziegler, 1966). These results were confirmed in several species of land plants and green algae (Anderson and Lim, 1972; Huber, 1978; Austin et al., 1992; Scagliarini et al., 1993; Baalmann et al., 1994). Wolosiuk and Buchanan (1978a) first demonstrated the increase of the NADP(H)-dependent GAPDH activity by reduced TRX, but the complex mechanism of GAPDH regulation involves also the inter-conversion between active tetramers and low activity oligomers (Pupillo and Piccari, 1973) and this change of quaternary structure is controlled by different ligands, including NAD(P)(H), ATP and BPGA (Pupillo and Piccari, 1973; O'brien et al., 1976; Wolosiuk and Buchanan, 1976; Cerff, 1978; Trost et al., 1993). *In vivo*, oligomers can be formed by GAPDH only (Pupillo and Piccari, 1973; Scheibe et al., 2002; Howard et al., 2011b), or include PRK as suggested by early works (Wara-Aswapati et al., 1980; Nicholson et al., 1987; Avilan et al., 1997). Later, it was understood that GAPDH-PRK complexes were actually assembled by a small TRX-regulated protein named CP12 (Wedel et al., 1997; see below).

**Figure 4:**
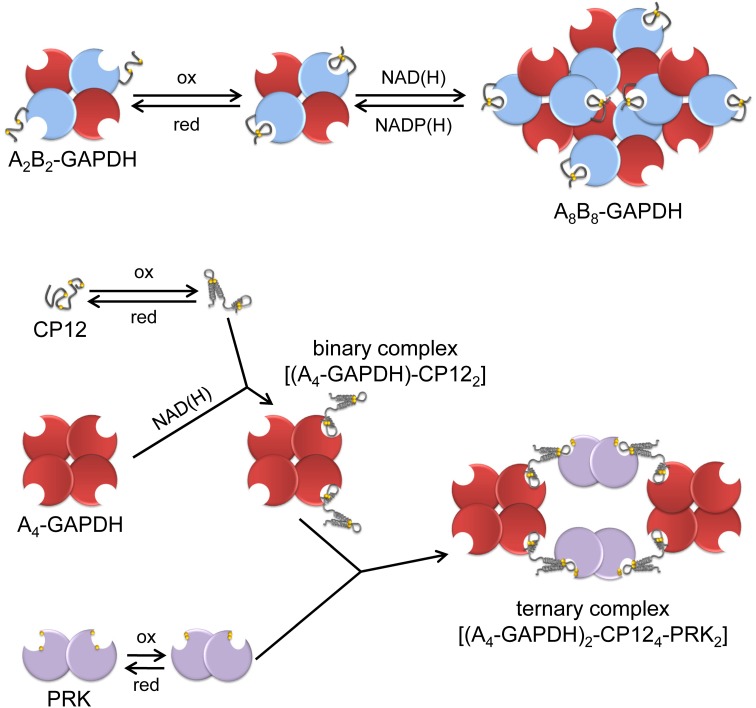
**A schematic view of the regulation and interactions of chloroplast A_4_-GAPDH, CP12, PRK and AB-GAPDH.** “Light conditions” (left side of the figure), consisting of increased levels of reduced TRXs and high NADP(H)/NAD(H) ratio, promote the reduction and dissociation of the supramolecular complexes. A_4_-GAPDH, reduced PRK and reduced A_2_B_2_-GAPDH are fully active in the light while reduced CP12 does not interact with partner proteins. “Dark conditions”(right side of the figure), consisting of decreased level of reduced TRXs and low NADP(H)/NAD(H) ratio, promote disulfide formation in PRK, CP12 and A_2_B_2_-GAPDH. These conditions promote the formation of the binary complex between A_4_-GAPDH and oxidized CP12 that further reacts with oxidized PRK forming the ternary complex A_4_-GAPDH/CP12/PRK. Concomitantly, A_2_B_2_-GAPDH undergoes oxidation and shifts to oligomeric form (A_8_B_8_-GAPDH). Within these complexes, the activity of both GAPDHs and PRK is strongly suppressed.

Photosynthetic GAPDH is coded by two types of genes (*gapA, gapB*) in Streptophytes [land plants and Charophytes (Petersen et al., 2006)], and Prasinophycean green algae [e.g., Ostreococcus, (Robbens et al., 2007)]. The *gapB* gene is absent in all other oxygenic phototrophs that usually contain a single *gapA* gene, except for cryptomonads, diatoms, and chromalveolates in general in which chloroplast GAPDH is encoded by *gapC*-type genes (Liaud et al., 1997). At the protein level, GAPA and GAPB are almost identical, but GAPB contains a specific C-terminal extension (CTE) of about 30 amino acids (Baalmann et al., 1996). The CTE contains the pair of Cys residues that are targeted by TRX and are responsible for the light/dark regulation of the enzyme (Sparla et al., 2002) (Figure 4). GAPA, or A-subunits, form stable homotetramers [A_4_-GAPDH, (Fermani et al., 2001)] that resemble the structure of glycolytic GAPDHs (Zaffagnini et al., 2013a,b, this series), or alternatively bind B-subunits in stoichiometric ratio to form heterotetramers (A_2_B_2_) and higher order oligomers [mainly A_8_B_8_; (Pupillo and Piccari, 1973)]. A_4_-GAPDH is a minor GAPDH isoform in higher plants (Scagliarini et al., 1993; Howard et al., 2011b), but is the only isoform of photosynthetic GAPDH in green algae, red algae and cyanobacteria (Petersen et al., 2006). Lacking the CTE, A_4_-GAPDH is not TRX-regulated *per se* but acquires this regulation through the interaction with the TRX-regulated proteins CP12 and PRK (Wedel and Soll, 1998; Graciet et al., 2004; Trost et al., 2006). This is the only known mechanism of light/dark regulation of GAPDH in green algae and cyanobacteria, and it will be discussed further below in the section on CP12. In chloroplasts of C_3_ plants, CTE- and CP12-based regulation of GAPDH co-exist (Scheibe et al., 2002), and CP12-assembled complexes may contain either A_4_- or A_2_B_2_-GAPDH (Carmo-Silva et al., 2011; Howard et al., 2011b). In C_4_ plants, the two systems appear instead to be separated: a proteomic study on maize revealed that CP12 is enriched in bundle sheath chloroplasts, together with GAPA and PRK, while GAPB is enriched in mesophyll chloroplasts (Majeran et al., 2005).

In land plants, the activity of AB-GAPDH isoforms primarily depends on the redox state of the two cysteines of the CTE [Cys349 and Cys358 in spinach (Sparla et al., 2002)]. The Cys349-Cys358 disulfide has a midpoint redox potential of −353 mV at pH 7.9 and is specifically reduced by TRXf (Marri et al., 2009). In the presence of reduced TRXf and at NADPH concentrations expected for illuminated chloroplasts, the NADPH-activity of AB-GAPDH is maximal and the enzyme is tetrameric (Scagliarini et al., 1993; Baalmann et al., 1994; Sparla et al., 2002). In this activated form, the 2′-phosphate group of NADPH interacts with Arg77 and Ser188, and these interactions are crucial for both coenzyme specificity [NADH contains an −OH in the 2′-position; (Falini et al., 2003)] and TRX-dependent regulation of GAPDH (Fermani et al., 2007). In fact, the disulfide bond between Cys349 and Cys358 shapes the CTE into a bulky hairpin structure that is harbored in proximity of the coenzyme-binding site. In this position, the negatively charged CTE distracts Arg77 from the 2′-phosphate of bound NADPH, with the consequence of depressing the NADPH-dependent activity, while leaving unaffected the NADH-dependent one. Both the crystal structure of oxidized A_2_B_2_-GAPDH (Fermani et al., 2007) and the kinetic characterization of site-specific mutants (Sparla et al., 2005) support this regulatory mechanism in which the CTE of GAPB acts as a redox-sensitive auto-inhibitory domain.

The CTE also controls the capability of A_2_B_2_-GAPDH to associate into the A_8_B_8_ isoform upon binding NAD(H) in place of NADP(H) (Figure 4). The effect is completely dependent on the CTE that must bear the Cys349-Cys358 disulfide (Baalmann et al., 1996; Sparla et al., 2002). Oligomeric AB-GAPDH has very low NADPH-activity and accumulates in chloroplasts in the dark (Scagliarini et al., 1993; Baalmann et al., 1994). Full recovery of GAPDH activity is obtained by reducing the CTE with reduced TRXf or by displacing oxidized CTE from the active site with ligands such as NADP(H), ATP, or BPGA (Trost et al., 2006). As recalled below, the CTE is homologous to the C-terminal half of CP12 and CP12 is engaged in protein-protein interactions with GAPA and PRK when containing a disulfide bond.

#### Phosphoribulokinase (PRK)

PRK catalyzes the phosphorylation of ribulose-5-phosphate to ribulose-1,5-bisphophate using ATP generated by thylakoid ATP synthase (Figure 1). The light-dependent activation of PRK was initially reported in the unicellular green alga Chlorella (Pedersen et al., 1966; Bassham, 1971) and later confirmed with isolated chloroplasts from spinach (Latzko et al., 1970; Avron and Gibbs, 1974). As in the case of GAPDH, the activation of PRK was found to be blocked by DCMU (Latzko et al., 1970; Avron and Gibbs, 1974; Champigny and Bismuth, 1976) and mimicked by DTT (Latzko et al., 1970; Anderson, 1973b; Anderson and Avron, 1976). Contrary to GAPDH, PRK has no cytosolic counterpart since this enzyme exclusively participates in the CBC. The enzyme is a homodimer in eukaryotes (Porter et al., 1986) and a homotetramer in cyanobacteria (Wadano et al., 1998). In anoxygenic photosynthetic prokaryotes PRK is octameric, but is not redox-regulated (Harrison et al., 1998). In plants, each PRK monomer contains 4 strictly conserved cysteines. PRK has a low activity in the oxidized form and is activated by TRX (Wolosiuk and Buchanan, 1978b). The molecular mechanism of PRK redox regulation was investigated on the spinach enzyme by chemical modification and site-directed mutagenesis of Cys16 and Cys55 that are located on the N-terminal part of the monomer and form a disulfide reduced by TRX (Porter and Hartman, 1988; Milanez et al., 1991; Brandes et al., 1996). Since Cys55 appears to play an important role in the catalysis by binding the sugar phosphate substrate (Porter and Hartman, 1990; Milanez et al., 1991), formation of the Cys16-Cys55 disulfide efficiently blocks the activity. The midpoint redox potential of PRK was found somehow variable between species [at pH 7.9: −315 mV in tomato (Hutchison et al., 2000); −330 mV in Arabidopsis (Marri et al., 2005); −349 mV in spinach (Hirasawa et al., 1999)], but tends to be less negative compared to that measured for other CBC enzymes [between −350 and −385 mV (Hirasawa et al., 1999, 2000; Hutchison et al., 2000; Sparla et al., 2002; Marri et al., 2005)]. Recently, the TRX specificity for Arabidopsis PRK activation was investigated and TRXf was found to be the most efficient compared to m-type TRXs, while no PRK reactivation was observed with x- and y-types TRXs (Marri et al., 2009). The three dimensional structure of plant PRK remains unknown precluding full understanding of the molecular mechanisms involved in the redox regulation mediated by TRX.

#### Fructose-1,6-bisphosphatase (FBPase)

FBPase catalyzes the dephosphorylation of fructose-1,6-bisphosphate (FBP) into fructose-6-phosphate (F6P) with the concomitant release of inorganic phosphate (Figure 1). The enzyme is a homotetramer of *ca.* 160 kDa. The cytosolic isoform of FBPase, which participates in gluconeogenesis, is not redox regulated by TRX. The light-dependent activation of FBPase was also initially reported in Chlorella (Pedersen et al., 1966; Bassham, 1971) and later confirmed in isolated chloroplasts from higher plants where the activation was found to be DCMU sensitive (Champigny and Bismuth, 1976; Kelly et al., 1976) and could be mimicked by DTT (Baier and Latzko, 1975). Oxidized FBPase has a basal activity (20–30%) and becomes fully activated upon disulfide reduction which is strictly dependent on TRXf, all other TRX types being inefficient. The molecular mechanism of TRX dependent activation of FBPase was initially unraveled for pea FBPase. Compared to its cytosolic counterparts, pea stromal FBPase contains two insertions of 5 and 12 amino acids containing 1 (Cys153) and 2 cysteines (Cys173 and Cys178), respectively. The mutation C153S or the double mutation C173S/C178S yielded a permanently active FBPase while the single mutant C173S and C178S retained a partial redox dependent regulation (Jacquot et al., 1995, 1997). These results suggested that the regulatory disulfide is formed between Cys153 and either with Cys173 or Cys178. The structure of pea FBPase revealed the presence of a unique Cys153-Cys173 disulfide, suggesting that the Cys153-Cys178 disulfide was only formed upon mutation of Cys173 although a more complex regulation implicating isomerization of disulfide bonds could not be completely ruled out (Chiadmi et al., 1999). Strikingly similar results were obtained for the 3 cysteines present in the insertions found in rapeseed FBPase (Rodriguez-Suarez et al., 1997). The midpoint redox potential of the regulatory disulfide bond was found to be −369 mV and −384 mV at pH 7.9 for pea and spinach FBPases, respectively (Hirasawa et al., 1999). Comparison of the structure of oxidized and reduced FBPase allowed understanding the conformational changes linking the redox state of the regulatory disulfide and the level of activation of the enzyme (Villeret et al., 1995; Chiadmi et al., 1999; Dai et al., 2004). Although the regulatory disulfide is at a distance of more than 20 Å from the active site, its formation forces a loop connecting two antiparallel beta strands to slide in toward the active site, thereby disrupting the binding sites for the catalytic Mg^2+^ cations (Chiadmi et al., 1999). Therefore, in light-regulated FBPase, the regulatory insertions that form the disulfide do not interact directly with the active site (like in malate dehydrogenase as described below) or in its proximity (like in AB-GAPDH), but stabilizes a general conformation in which the active site is almost non-functional.

#### Sedoheptulose-1,7-bisphosphatase (SBPase).

SBPase catalyzes the dephosphorylation of sedoheptulose-1,7-bisphosphate (SBP) into sedoheptulose-7-phosphate (S7P) with the concomitant release of inorganic phosphate (Figure 1). SBPase is a homodimer of *ca.* 70 kDa that is unique to the CBC and has no cytosolic counterpart. SBPase is found in all photosynthetic eukaryotes but not in cyanobacteria which encode a bifunctional FBPase possessing also SBPase activity (Tamoi et al., 1996). As in the case of GAPDH and FBPase, the light-dependent activation of SBPase was also initially reported in Chlorella (Pedersen et al., 1966; Bassham, 1971), confirmed in isolated chloroplasts from land plants and found to be mimicked by DTT (Anderson, 1974; Schürmann and Buchanan, 1975; Anderson and Avron, 1976). By contrast with other CBC enzymes, the oxidized form of SBPase is completely inactive and its reactivation absolutely requires the TRX-mediated light activation. SBPase redox regulation has only been analyzed for the wheat enzyme that possesses 7 cysteines among which 4 are strictly conserved in all organisms. Site-directed mutagenesis of the wheat enzyme suggested the existence of a single regulatory disulfide between Cys52 and Cys57 (Dunford et al., 1998). However, in this study, the activity of recombinant wheat mutant SBPase was only measured in *E. coli* crude extracts using DTT as electron donor that may have mediated SBPase activation through *E. coli* TRXs. Therefore, the molecular mechanism underlying the redox regulation of SBPase remains to be clearly established with the purified enzyme. The enzyme from maize leaves was reported to be, like FBPase, specifically activated by TRXf (Nishizawa and Buchanan, 1981) but not all TRX types have yet been tested. SBPase sequences share a significant homology with FBPase, possibly due to a common origin (Raines et al., 1992; Martin et al., 1996). Therefore, although the structure of plant SBPase remains undetermined, a model has been proposed based on the structure of pig FBPase (Dunford et al., 1998). This model suggested that like in FBPase, the reduction of SBPase disulfide would trigger its activation through a general conformational change of the enzyme that shapes the active site.

### Other TRX-dependent enzymes linked to the Calvin–Benson cycle

#### NADP-dependent malate dehydrogenase (NADP-MDH)

**Figure 5:**
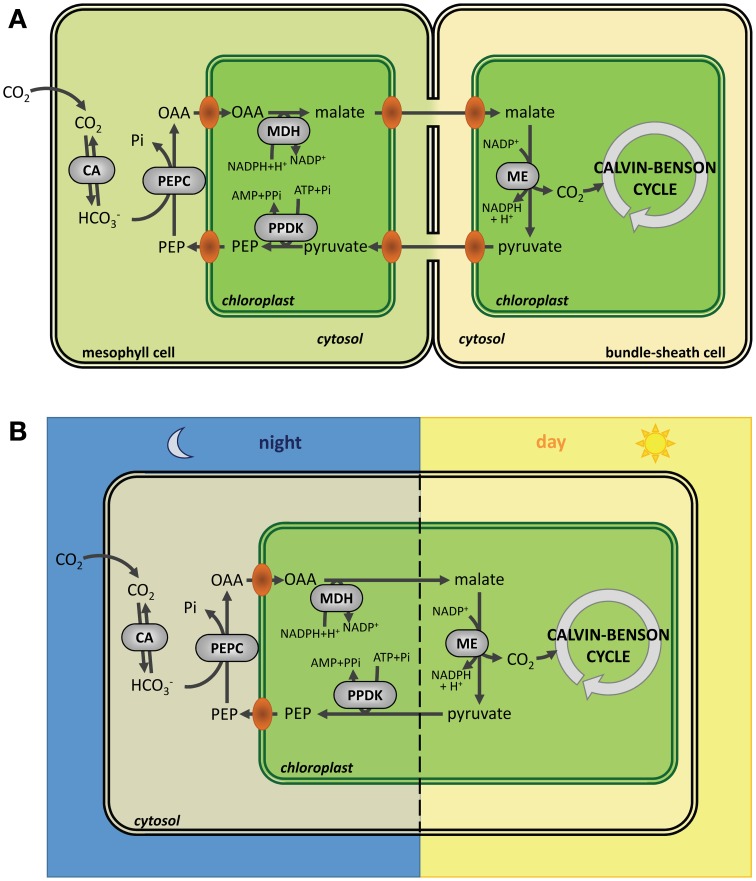
**Carbon fixation in C4 and CAM plants. (A)** Carbon fixation in C4 plants. Compared to C3 plants, carbon fixation in C4 plants occurs in two steps: CO_2_ is first fixed by PEPC (phosphoenolpyruvate carboxylase) in mesophyll cells to form oxaloacetate (OAA) that is converted to malate by NADP-MDH (NADP-malate dehydrogenase) before export to bundle sheath cells were CO_2_ is released by ME (malic enzyme). Finally, CO_2_ is fixed by Rubisco. **(B)** Carbon fixation in CAM plants. In CAM plants, carbon fixation is split in two steps, but, compared to C4 plants, this fixation is separated in time instead of being separated in space with cells fixing CO_2_ by PEPC (phosphoenolpyruvate carboxylase) and accumulating malate during the night before release of CO_2_ by ME (malic enzyme) and fixation by Rubisco during the day. CA, carbonic anhydrase; PEPC, phosphoenolpyruvate carboxylase; MDH, NADP malate dehydrogenase; ME, malic enzyme; PPDK, pyruvate, phosphate dikinase; OAA, oxaloacetate; PEP, phosphoenolpyruvate.

**Figure 6:**
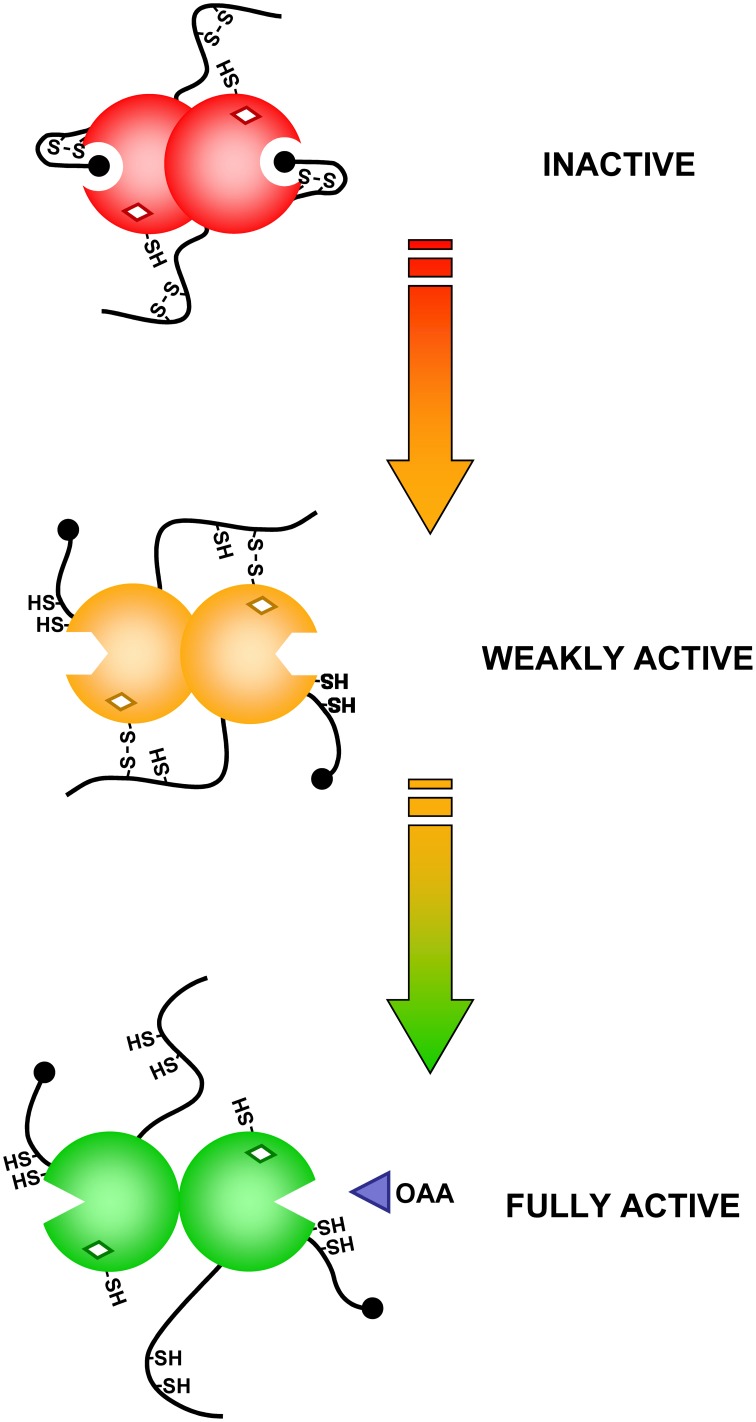
**Schematic representation of the activation mechanism of Sorghum NADP-MDH**.

NADP-MDH catalyzes the reduction of oxaloacetate (OAA) into malate using NADPH as electron donor. The enzyme is a homodimer of *ca*. 70 kDa. NADP-MDH plays a key role for CO_2_ fixation in C4 and CAM plants where photorespiration, linked to the oxygenase activity of Rubisco, is limited through an alternative CO_2_ fixation pathway initiated by phosphoenolpyruvate carboxylase (PEPC) (Foyer et al., 2009) (Figure 5). CO_2_ fixation by PEPC yields oxaloacetate that is converted to malate by NADP-MDH. The malic enzyme converts malate into phosphoenolpyruvate and CO_2_ in the proximity of Rubisco. These carbon concentration mechanisms limit photorespiration and ensure optimal photosynthesis efficiency under specific growth conditions. In C3 plants, NADP-MDH is involved in the export of reducing power, in the form of malate, from the stroma to the cytosol through the malate valve. Light-dependent activation of NADP-MDH was initially reported in C4 plants (Johnson and Hatch, 1970) and later confirmed in C3 plants (Johnson, 1971). As in the case of CBC enzymes, the light-dependent activation was confirmed on isolated chloroplasts, found to be blocked by DCMU and mimicked by DTT (Anderson, 1973a; Anderson and Avron, 1976). NADP-MDH is probably one of the best studied light-regulated enzyme in the chloroplast both at the molecular and structural levels. By contrast with most CBC enzymes which exhibit a basal activity in the oxidized form, oxidized NADP-MDH is totally inactive and strictly dependent on light-driven TRX activation. The molecular mechanism of NADP-MDH activation has been initially unraveled on the enzyme from the C4 plant *Sorghum bicolor*. Compared to the redox independent NAD-dependent MDH isoforms, Sorghum NADP-MDH possesses N- and C-terminal extensions and contains 8 cysteine residues strictly conserved in land plants. Site-directed mutagenesis and chemical modifications allowed understanding the molecular mechanism of TRX-dependent activation of this enzyme (reviewed in Miginiac-Maslow and Lancelin, 2002). Two regulatory disulfides, located in each extension, are present in the oxidized form and have to be both reduced by TRX for full activation of the enzyme. Reduction of the Cys24-Cys29 N-terminal disulfide allows a slow conformational change of the enzyme while reduction of the Cys365-Cys377 C-terminal disulfide is required to give access to the active site (Issakidis et al., 1992, 1993, 1994, 1996; Lemaire et al., 1994). Indeed, in the oxidized form, the penultimate glutamate residue interacts with the active site Arg204 thereby blocking access of OAA (Ruelland et al., 1997; Hirasawa et al., 2000). In addition, the internal Cys207 can form a TRX-reducible disulfide with Cys24, suggesting that disulfide isomerization may be required during activation (Ruelland et al., 1997; Hirasawa et al., 2000). These results were later found to be consistent with the structures of Sorghum and Flaveria NADP-MDH (Carr et al., 1999; Johansson et al., 1999) and have allowed to propose a detailed model of the molecular mechanism of activation of NADP-MDH (Figure 6). The standard redox potentials of the two regulatory disulfides are not equivalent, the N-terminal disulfide (*E*_*m*_ = −344 mV at pH 7.9) being more positive, and therefore easier to reduce, than the C-terminal disulfide (*E*_*m*_ = −384 mV at pH 7.9) that will require an excess of reduced TRX for its reduction. This difference suggests the existence of a sequential activation: reduction of the N-terminal disulfide would occur first and allow “pre-reduction” of the enzyme in a form that can be rapidly activated when the reducing power of the chloroplast reaches a threshold level. The knowledge acquired on the redox regulation of Sorghum NADP-MDH allowed transforming the constitutively active NAD-MDH from the thermophilic bacteria *Thermus flavus* into a TRX-dependent enzyme by grafting of the Sorghum N- and C-terminal extensions (Issakidis-Bourguet et al., 2006).

Interestingly, Chlamydomonas NADP-MDH was found to also possess N- and C-terminal extensions but the N-terminal extension does not contain any cysteine. The enzyme is also strictly dependent on TRX-mediated activation through reduction of a single regulatory disulfide located in its C-terminal extension (Lemaire et al., 2005). The redox potential of this disulfide (*E*_*m*_ = −369 mV at pH 7.9) is more positive than the C-terminal disulfide of Sorghum NADP-MDH (*E*_*m*_ = −384 mV pH 7) and can be reduced by both TRXm and TRXf1 from Chlamydomonas. Conversely, Chlamydomonas TRXf1 is not able to activate Sorghum NADP-MDH, suggesting structural and thermodynamic differences between algal and land plants TRXf. Analysis of different Sorghum NADP-MDH mutants suggested that the redox potential of the algal TRXf is significantly lower than that of land plant TRXf (Lemaire et al., 2005). This surprising result suggests the existence of a coevolution of the redox properties of TRXs and NADP-MDH. From an evolutionary point of view, the redox regulation of Chlamydomonas NADP-MDH appears like a first step toward the complex regulation existing in land plants. The requirement for such a sophisticated control may be linked to the multicellular nature of land plants where malate is a circulating form of reducing power (Scheibe, 2004) while in Chlamydomonas malate is only exchanged between the unique chloroplast and the cytosol. These results also suggest that the redox regulatory sequences have been progressively added to non-regulated enzymes during the course of evolution.

#### Rubisco activase

Rubisco activase is a molecular chaperone of the AAA+ family that uses the energy from ATP hydrolysis to release tight binding inhibitors from the active site of Rubisco (reviewed in Portis, 2003; Portis et al., 2008). The ATPase activity of Rubisco activase is controlled by the ADP/ATP ratio and/or by the Fd/TRX system. In many species, such as Arabidopsis, two isoforms of activase are present: a short form (beta isoform) and a long form (alpha isoform). The two forms are either generated by alternative splicing or encoded by distinct genes (Werneke and Ogren, 1989; Salvucci et al., 2003; Yin et al., 2010). Compared to the beta isoform, the alpha isoform differs by the presence of a C-terminal extension containing two conserved cysteines. In Arabidopsis, site-directed mutagenesis revealed that these residues, Cys392 and Cys411, form a disulfide reduced by TRXf in the light (Zhang et al., 2001). The extension contains negative charges that interact with the ATP binding site (Wang and Portis, 2006; Portis et al., 2008). Indeed, the oxidized enzyme has a decreased affinity for ATP and is inhibited by ADP while reduction by TRXf alleviates this inhibition. This regulation controls the activity of both alpha and beta isoforms in the holoenzyme (Zhang et al., 2001). Some species such as tobacco, maize, or green algae only contain the short beta isoform but still exhibit light dependent regulation of Rubisco activase activity (Salvucci et al., 1987). A recent study revealed that tobacco beta isoform, by contrast with the beta isoform from Arabidopsis, has a unique sensitivity to ADP/ATP ratios that is responsible for the light regulation of the activity (Carmo-Silva and Salvucci, 2013). Several studies have concluded that Rubisco activase forms hexamers in solution, and that this may be the active form (Keown et al., 2013; Mueller-Cajar et al., 2013). The structure of tobacco Rubisco activase forms a helical arrangement in the crystal structure, with six subunits per turn (Stotz et al., 2011). However, Rubisco activase appears to form a wide range of structures in solution, ranging from monomers to oligomers, and an open spiraling structure rather than a closed hexameric structure has been recently proposed (Keown et al., 2013). Numerous features of Rubisco activase are reminiscent of GAPDH which also can assemble into higher oligomeric states and has both redox-regulated (GAPB) and non-redox-regulated (GAPA) subunits that differ by a C-terminal extension containing a TRX-reduced regulatory disulfide that exerts control on the activity of the mixed oligomer (A_n_B_n_-GAPDH).

#### CP12

CP12 was discovered by serendipity in higher plants as a novel protein of 78 amino acids with a C-terminal sequence homologous to the CTE of GAPB subunits of GAPDH (Pohlmeyer et al., 1996). CP12 was then found to be widespread in oxygenic photosynthetic organisms, including cyanobacteria, and GAPB subunits of land plants are now believed to be the result of a gene fusion event between GAPA and CP12 that must have occurred at the origins of Streptophytes (Petersen et al., 2006) or before (Robbens et al., 2007).

Most CP12s contain four conserved cysteines able to form two disulfide bonds (Groben et al., 2010; Marri et al., 2010; Stanley et al., 2013). CP12 from both *Synechococcus* sp. PCC7942, Chlamydomonas and Arabidopsis were the most extensively studied. The protein is intrinsically disordered, particularly so when it is fully reduced, but still poorly structured when it bears both disulfide bonds (Graciet et al., 2003; Marri et al., 2008, 2010). In Arabidopsis, the C-terminal disulfide has redox properties similar to GAPB disulfide [*E*_*m*_ = −352 mV at pH 7.9, (Marri et al., 2008)] and is reduced by TRXs, though with no strict specificity (Marri et al., 2009). Oxidized CP12 binds to A_4_-GAPDH more tightly than CTE through interactions with both the bound coenzyme and the catalytic site of the enzyme. (Fermani et al., 2007, 2012; Matsumura et al., 2011). In Arabidopsis (Marri et al., 2008; Fermani et al., 2012) and Chlamydomonas (Kaaki et al., 2013), A_4_-GAPDH binds two CP12, while four CP12 are bound to A_4_-GAPDH in Synechococcus (Matsumura et al., 2011). CP12 binding is very strong in Chlamydomonas (k_*D*_ 0.4 nM) and causes inhibition of GAPDH activity (Erales et al., 2011), whereas in Arabidopsis CP12 binding is much weaker [k_D_ 0.2 μM, (Marri et al., 2008)] and inhibition is negligible (Marri et al., 2005, 2008). However, in these and other organisms, the binary complex GAPDH/CP12 can then bind PRK forming the GAPDH/CP12/PRK ternary complex, in which both enzyme activities are strongly down-regulated (Marri et al., 2005). NAD(H) binding to GAPDH is an absolute requirement for complex formation because the 2′-phosphate of NADPH sterically hinders the attachment of CP12 (Matsumura et al., 2011; Fermani et al., 2012). Dissociation of the complex, and recovery of enzyme activity is rapidly obtained by reduced TRXs (Marri et al., 2009), but also by BPGA, NADPH or ATP that displace CP12 from its binding sites on GAPDH and PRK, respectively (Wedel et al., 1997; Scheibe et al., 2002; Graciet et al., 2004; Marri et al., 2005; Tamoi et al., 2005; Howard et al., 2008). CP12-assembled complexes of GAPDH and PRK accumulate in the dark, both in cyanobacteria and in chloroplasts, probably favored by the oxidation of the TRX pool and by low NADP(H)/NAD(H) ratios (Scheibe et al., 2002; Tamoi et al., 2005; Howard et al., 2008, 2011b). In Synechococcus, inactivation of *cp12* gene impaired cell growth in normal light-dark cycles, but not under continuous illumination, supporting the role of CP12 in light-dark regulation of the CBC in this organism (Tamoi et al., 2005). CP12 is also coded by the small genome of cyanophages that infect marine cyanobacteria including Synechococcus, apparently with the function of redirecting the carbon flux of the prokaryote from the CBC to the pentose phosphate pathway, thereby sustaining NADPH production for phage replication (Thompson et al., 2011). However, other functions of CP12 have been proposed, particularly in land plants like tobacco, where antisense suppression of CP12 severely restricts growth and alters carbon partitioning through mechanisms that are still poorly understood (Howard et al., 2011a).

### Evolution of regulatory sequences

**Figure 7:**
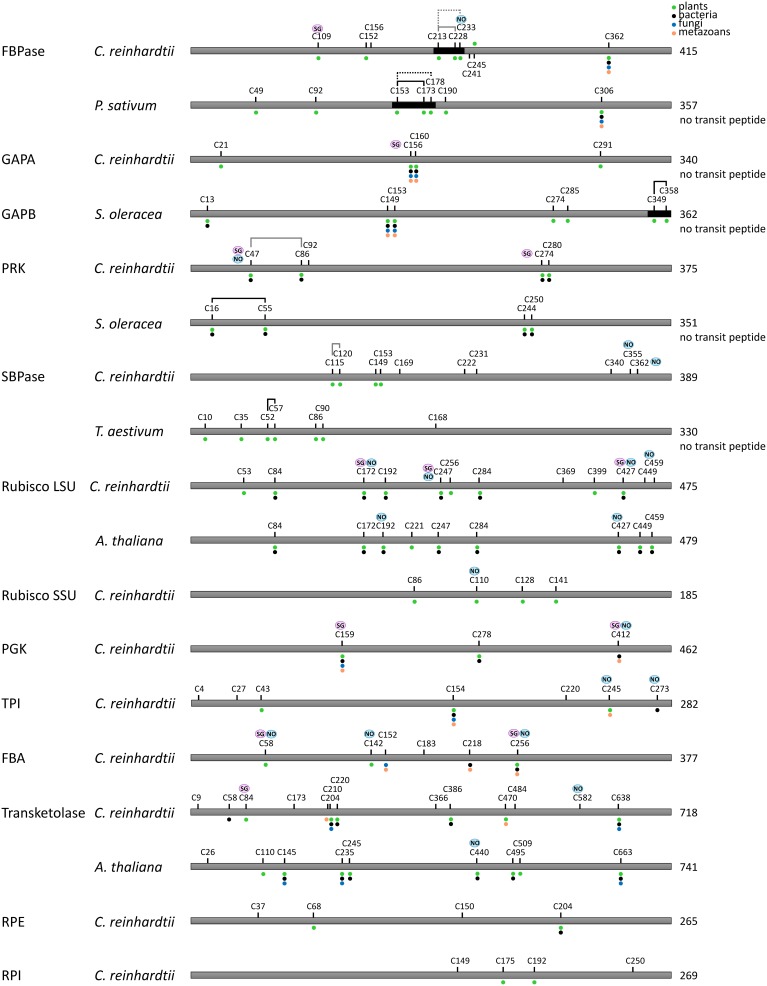
**Redox regulated cysteines in Calvin–Benson cycle enzymes.** All proteins are schematically represented linearly. The numbering corresponds to the sequence with the transit peptide, except when “no transit peptide” is indicated on the right. Lines between cysteines indicate confirmed (black) or suggested (black and dashed) disulfide bonds, whereas gray lines indicate putative regulatory disulfides in *C. reinhardtii* by homology with the disulfide identified for other species. Cysteines identified as nitrosylated and glutathionylated are labeled with NO (blue) and SG (purple), respectively. The dots indicate cysteine conservation in photosynthetic organisms (green), bacteria (black), fungi (blue) and metazoans (orange). Dark boxes correspond to insertions/extension present in the TRX-regulated isoforms and absent in the isoforms not regulated by TRX.

Comparison of the redox regulatory properties of enzymes from cyanobacteria, diatoms, algae and higher plants suggest that the light-dependent regulation mediated by TRX has been progressively introduced during evolution (Ruelland and Miginiac-Maslow, 1999; Lemaire et al., 2007) (Figure 7). Comparison with non-redox-regulated forms suggest that regulatory sequences have been grafted within N- or C-terminal extensions (GAPDH, NADP-MDH, Rubisco Activase) or inserted in the sequence (FBPase). For some enzymes, there is no obvious insertion or extension but this mainly applies to enzymes unique to the CBC (PRK, SBPase). Interestingly, in the diatom *O. sinensis* PRK contains the regulatory cysteines but the redox potential of the disulfide is less negative than in PRK from higher plants, suggesting that the enzyme might not be regulated by TRX *in vivo* (Michels et al., 2005). In a survey on different algal groups, redox-regulation of PRK was found to be greatest in chlorophytes, but low or absent in a red alga and most chromalveolates (including diatoms), and linked to the number of amino acids separating the two regulatory cysteine residues (Maberly et al., 2010). Several other enzymes in diatoms may also be TRX independent due to the absence of regulatory cysteines such as NADP-MDH (Ocheretina et al., 2000) or GAPDH (Liaud et al., 2000).

## Proteomics unravel new redox-dependent regulations

Recent advances in the field of proteomics and genomics associated with our increasing understanding of redox post-translational modifications have considerably challenged our current models of the redox dependent regulation of the CBC described in the above sections. These new data suggest that all enzymes of the CBC are regulated by a complex network of redox PTMs that is only starting to emerge. The following sections will describe these new developments and discuss their potential physiological and cellular importance.

### Thioredoxin targets

As described above, the availability of plant genome sequences revealed the existence of an unsuspected multiplicity of TRXs. At the beginning of the 2000s, the number of TRXs was even higher than the number of known TRX-regulated targets. This multiplicity raised questions about the specificity of the different TRX isoforms for their target enzymes. Moreover, systematic biochemical analysis of the ability of Arabidopsis TRX isoforms to activate different chloroplastic TRX targets revealed that TRXs are not equivalent and exhibit strong specificities (Collin et al., 2003, 2004). This suggested that additional TRX targets probably remained to be identified and prompted several groups to develop new proteomic-based strategies to identify these unrecognized targets (reviewed in Lemaire et al., 2007; Schürmann and Buchanan, 2008; Lindahl et al., 2011). Two main strategies have been employed. The most common is based on the ability of a monocysteinic TRX to form covalent heterodimers with its target enzymes. Indeed, studies on the reaction mechanism allowing the reduction of an oxidized target by a reduced TRX had established that the most N-terminal cysteine of TRX active site performs an initial nucleophilic attack leading to the formation of a transient mixed-disulfide between the TRX and the target. This mixed-disulfide is then immediately reattacked by the second cysteine of the active site to yield an oxidized TRX and a reduced target. Consequently, mutating the second cysteine of the active site allows stabilization of the mixed-disulfide. TRX-affinity columns, based on a resin-bound monocysteinic TRX, have been employed to trap TRX targets which can be eluted by DTT reduction and identified by proteomic analysis. Many targets have been identified using this type of affinity columns (Motohashi et al., 2001; Goyer et al., 2002; Balmer et al., 2003, 2004b, 2006b; Lindahl and Florencio, 2003; Lemaire et al., 2004; Wong et al., 2004; Yamazaki et al., 2004; Hosoya-Matsuda et al., 2005; Marchand et al, 2006; Marchand et al., 2010; Pérez-Pérez et al., 2006, 2009). In addition, affinity columns based on wild-type TRX have also been used to detect proteins interacting electrostatically with TRX (Balmer et al., 2004a). The second most widely used approach is based on the visualization of proteins reduced by TRX *in vitro* by specific labeling of exposed thiols by fluorescent probes like monobromobimane (mBBr) (Yano et al., 2001; Marx et al., 2003; Wong et al., 2003, 2004; Balmer et al., 2006a,b; Yano and Kuroda, 2006; Hall et al., 2010) or Cy5 maleimide (Maeda et al., 2004) or by radioactive probes like ^14^C-iodoacetamide (Marchand et al., 2004; Marchand et al, 2006). A comparison between both methods suggested that they are complementary since only a partial overlap is found between the different targets identified (Marchand et al, 2006).

All these proteomic studies have allowed identifying more than 300 putative TRX targets from the cyanobacteria *Synechocystis* sp. PCC6803, the unicellular green alga *Chlamydomonas reinhardtii* and numerous higher plant species (reviewed in Michelet et al., 2006; Lindahl et al., 2011). These proteomic methods are therefore powerful but they also suffer from a lack of specificity. Indeed, proteomic approaches with different types of TRX yielded roughly the same targets while a strong or exclusive specificity of most targets for a specific TRX type is generally observed *in vitro*. For example, columns based on monocysteinic TRXf and TRXm basically retain the same targets while FBPase and GAPDH are exclusively activated by TRXf and not by TRXm (Collin et al., 2003; Marri et al., 2009). This suggests that monocysteinic TRX have peculiar properties distinct from the WT enzyme and/or that the loss of specificity is due to the use of high concentrations of TRX. Indeed, while enzymes show a preference for some TRX types at physiological TRX concentration, many TRX types become able to significantly activate a number of TRX targets if used at high concentration (Collin et al., 2003, 2004). By contrast, the diversity of the targets appeared to be strongly dependent on the type of protein extracts employed (organism, tissue, growth conditions…). The problem of specificity is even larger since monocysteinic GRX columns also retain the same type of targets than those bound on TRX columns (Rouhier et al., 2005; Li et al., 2007). Classical GRXs belong to the TRX family and contain an active site disulfide (Cys-Pro-Trp/Phe-Cys) that is reduced by glutathione (Rouhier et al., 2008; Zaffagnini et al., 2012c). GRXs can reduce disulfide bonds on their target proteins, although much less efficiently than TRX, but they are thought to play a more prominent role in the control of protein (de)glutathionylation (Zaffagnini et al., 2012c).

Among all putative TRX targets, more than 130 are located in chloroplasts (Lemaire et al., 2007; Lindahl and Kieselbach, 2009). The well-established targets of TRX participating directly or indirectly in the CBC were recovered by proteomic approaches (FBPase, SBPase, GAPDH, PRK, CP12, Rubisco activase). More surprisingly, all other enzymes of the CBC were also identified among putative targets, suggesting that they might all be redox regulated (Table 1). All CBC enzymes were, however, not identified within the same study, possibly because of a low coverage rate due to the use of 2D-gels and MALDI-TOF mass spectrometry in most studies. This suggests that the number of TRX targets could be significantly higher than presently known and that the combination of TRX-affinity chromatography or thiol-labeling with modern gel-free proteomic methods may reveal a much greater diversity of new potential targets of TRXs.

**Table 1:** **Summary of redox proteomic analyses of Calvin–Benson cycle enzymes and related proteins**.

	**Putative nitrosylation targets (references)**	**Putative nitrosylated cysteine (Cys numbering)**	**Putative glutathionylation targets (references)**	**Putative glutathionylated cysteine (Cys numbering)**	**Putative thioredoxin targets (references)**	**Putative glutaredoxin targets (references)**
Rubisco S	2, 3, 5, 6, 8, 11, 12, 26	C^64^	1		10, 13, 14, 15, 16, 20	17
Rubisco L	2, 3, 4, 5, 6, 7, 8, 12, 26	C^172/247/427/459^/*C*^*192/427*^	1	C^172/247/427^	10, 15, 16, 19, 20, 22, 23	17
PGK	2, 6, 8	C^411^	1, 9	C^411/C158^	16, 21	17
GAPDH A	2, 3, 5, 7, 8, 11		1		13, 16, 18, 20, 22	17
GAPDH B	5, 8				14, 16	
TPI	2, 3, 5, 8, 11	C^245/273^			14, 18, 20	17
FBA	2, 3, 5, 6, 8, 12, 26	C^58/142/256^	1, 25	C^58/256^	10, 15, 22, 24	17
FBPase	2	C^233^	1	C^109^	16, 18, 22	17
Transketolase	2, 4, 5, 8, 11, 12	C^582^/*C*^440^	1	C^84^	14, 16, 18, 20, 21, 22	17
SBPase	2, 8, 12	C^355/362^	1		10, 13, 14, 18, 20	
RPE	2		1		14, 18, 20	
RPI	2, 3		1, 9		10, 18, 20	
PRK	2, 3, 6, 7, 8, 11	C^47^	1	C^47/274^	10, 14, 18, 20, 22	17
CP12^*^	3				18, 20	
NADP-MDH	2	C^74^	1		14, 16, 18, 20	
Rubisco Activase	2, 3, 4, 6, 8, 11	C^196/289^/*C*^175^	1		13, 14, 15, 16,18, 20	17

Calvin–Benson cycle enzymes are listed in the order of the reactions starting with Rubisco. The three last enzymes participate indirectly in the regulation of the cycle. The references correspond to proteomic studies that allowed identification of the enzyme as nitrosylated, as glutathionylated or as thioredoxin or glutaredoxin targets. Modification sites identified by proteomic methods are listed with the numbering of the cysteines identified as glutathionylated (Zaffagnini et al., 2012a) and nitrosylated (Morisse et al. manuscript in preparation) in Chlamydomonas reinhardtii in normal text and the cysteines found as nitrosylated in Arabidopsis thaliana (Fares et al., 2011) in italics. Numberings correspond to the full-length protein with its transit peptide. References: 1, (Zaffagnini et al., 2012a); 2, Morisse et al. manuscript in preparation; 3, (Lin et al., 2012); 4, (Fares et al., 2011); 5, (Lindermayr et al., 2005); 6, (Kato et al., 2013); 7, (Romero-Puertas et al., 2008); 8, (Tanou et al., 2012); 9, (Michelet et al., 2008); 10, (Lemaire et al., 2004); 11, (Tanou et al., 2009); 12, (Abat and Deswal, 2009); 13, (Motohashi et al., 2001); 14, (Balmer et al., 2003); 15, (Balmer et al., 2006b); 16, (Balmer et al., 2004a); 17, (Rouhier et al., 2005); 18, (Marchand et al., 2004); 19, (Lindahl and Florencio, 2003); 20, (Marchand et al, 2006); 21, (Balmer et al., 2006a); 22, (Pérez-Pérez et al., 2009); 23, (Hall et al., 2010); 24, (Marchand et al., 2010); 25, (Ito et al., 2003); 26, (Abat et al., 2008).

Nevertheless, the identification of all CBC enzymes as potential targets of TRXs suggested the existence of a complex redox control of these enzymes that may be much more sophisticated than the light-dependent regulation initially uncovered for four enzymes of the cycle. These features may allow a fine tuning of the Calvin–Benson cycle in response to environmental changes that affect ROS production and the intracellular redox state. There are two non-mutually exclusive possibilities to explain this surprising result. First, all the CBC enzymes may contain a TRX-reducible disulfide bond, like the four established TRX-targets. These regulations may have been initially missed, for example, because of their low activation upon reduction (e.g., below 50%) or because the disulfide controls enzyme properties that have not been investigated (protein stability, cooperativity between subunits, protein-protein interactions, etc.). This may be the case for phosphoglycerate kinase (PGK) which was shown to be redox regulated, possibly by TRX, in *Phaeodactylum tricornutum* (Bosco et al., 2012) and *Synechocystis* sp. PCC6803 (Tsukamoto et al., 2013). To date, none of these putative TRX dependent redox regulations have been confirmed experimentally on any other CBC enzyme. The second possibility is that these cysteines are not attacked (or reduced) by TRX because they are engaged in a disulfide bond but because they harbor another type of cysteine oxidative modification. Indeed, although TRX are efficient protein disulfide reductases, they are also playing a role in the control of other post-translational modifications including sulfenylation, nitrosylation or glutathionylation. Therefore, putative TRX targets identified by proteomic approaches may represent proteins containing diverse types of redox PTM and should therefore be considered as putative redox regulated proteins rather than proteins containing a TRX-reducible disulfide. This was demonstrated for Chlamydomonas isocitrate lyase, an enzyme of the glyoxylate cycle participating in acetate assimilation which was retained on a monocysteinic TRX affinity column (Lemaire et al., 2004). Detailed biochemical analysis of this enzyme revealed that the enzyme is strongly and reversibly inhibited by glutathionylation but does not contain any TRX-reducible disulfide bond (Bedhomme et al., 2009). We recently obtained comparable results with the Calvin–Benson enzyme triosephosphate isomerase (TPI) which does not appear to contain a regulatory disulfide but was found to undergo glutathionylation and nitrosylation *in vitro* (Zaffagnini et al., 2013a). Although the number of studies is more limited, mounting evidence suggests that glutathionylation and nitrosylation also control enzymes of the CBC. These recent developments are detailed in the next sections.

### Multiple redox post-translational modifications

During the last decade, glutathionylation and nitrosylation have emerged as crucial PTMs playing a major role in numerous fundamental cell processes, especially cell signaling pathways (Hess et al., 2005; Besson-Bard et al., 2008; Mieyal et al., 2008; Rouhier et al., 2008; Dalle-Donne et al., 2009; Foster et al., 2009; Astier et al., 2011; Hess and Stamler, 2012; Zaffagnini et al., 2012c).

Glutathione is the major low molecular weight antioxidant in most species and exists in the reduced (GSH) and oxidized (GSSG) forms. GSH is the major form due to constant reduction of GSSG to GSH by glutathione reductase (GR) at the expense of NADPH. Glutathione is considered as a major cellular antioxidant and redox buffer but also plays an important role in a myriad of cellular and physiological functions including detoxification of heavy metals and xenobiotics, root growth or pathogen responses (Noctor et al., 2012). Glutathionylation is a post-translational modification triggered by oxidative stress conditions and consisting of the formation of a mixed-disulfide between a protein free thiol and the thiol of a molecule of glutathione. Although the precise mechanism leading to glutathionylation is still unclear *in vivo*, it is considered to occur mainly either through reactive oxygen species (ROS)-dependent sulfenic acid formation followed by reaction with reduced glutathione (GSH) or by thiol/disulfide exchange with oxidized glutathione (GSSG). The reverse reaction, named deglutathionylation, is mainly catalyzed by GRXs.

Nitrosylation consists in the formation of nitrosothiols by reaction of protein thiols with nitric oxide (NO). It can be triggered chemically by reactive nitrogen species (RNS) which includes NO and its related species (such as the nitrosonium cation, NO^+^; nitroxyl anion, NO^−^; dinitrogen trioxide, N_2_O_3_ or peroxynitrite, ONOO^−^) but also by transnitrosylation reactions mediated by small nitrosothiols (e.g., nitrosoglutathione, GSNO) or by other nitrosylated proteins (Hogg, 2002; Hess et al., 2005; Benhar et al., 2009; Zaffagnini et al., 2013b). The reduction of nitrosothiols on proteins, i.e., denitrosylation, entails two possible mechanisms dependent on reduced glutathione (GSH) or reduced TRX (Benhar et al., 2009; Sengupta and Holmgren, 2013).

To date, several hundreds of targets of glutathionylation and nitrosylation have been identified in bacteria, yeast, animals and plants, suggesting a role for these redox modifications in many cellular processes (Mieyal et al., 2008; Astier et al., 2011; Hess and Stamler, 2012; Zaffagnini et al., 2012c; Maron et al., 2013). In our two recent studies, the use of biotin-based enrichment strategies using streptavidin affinity chromatography combined with up-to date mass spectrometry instruments allowed identification of 225 glutathionylated proteins and 492 nitrosylated proteins in Chlamydomonas (Zaffagnini et al., 2012a; Morisse et al. manuscript in preparation). There is a striking overlap between potential TRX targets and proteins identified as nitrosylated or glutathionylated through proteomic studies. This suggests that these proteins are regulated by multiple redox PTMs or that the methods aimed at identifying TRX targets also identified nitrosylated and glutathionylated proteins. The latter possibility is consistent with the fact that TRX was proposed to catalyze both (de)nitrosylation and (de)glutathionylation reaction for some targets (Benhar et al., 2008; Greetham et al., 2010; Bedhomme et al., 2012; Zaffagnini et al., 2013b).

Notably, all CBC enzymes appear to be modified by glutathionylation (Table 1). Proteomic studies initially reported *in vivo* glutathionylation for fructose-1,6-bisphosphate aldolase (FBA) in Arabidopsis (Ito et al., 2003) and for phosphoglycerate kinase (PGK) and ribose-5-phosphate isomerase (RPI) in Chlamydomonas (Michelet et al., 2008). More recently, all CBC enzymes were found to undergo glutathionylation in Chlamydomonas (Zaffagnini et al., 2012a, 2013a). The modifications of FBA, PGK and A_4_-GAPDH from Chlamydomonas were confirmed by demonstrating that the purified protein is glutathionylated after treatment with BioGSSG (biotinylated oxidized glutathione) *in vitro*. Chlamydomonas PRK was found to be strongly inhibited by GSSG and the activity could be fully recovered after DTT treatment (Zaffagnini et al., 2012a). The glutathionylation of different isoforms of GAPDH from Arabidopsis was investigated in detail (Zaffagnini et al., 2007). Arabidopsis A_4_-GAPDH was shown to be glutathionylated *in vitro* on its catalytic cysteine with a concomitant loss of enzyme activity. The enzyme is very sensitive to oxidants and is rapidly and irreversibly inactivated by H_2_O_2_. However, incubation of the enzyme with H_2_O_2_ in the presence of GSH leads to glutathionylation, most likely through a mechanism involving a sulfenic acid intermediate. Therefore, glutathionylation efficiently protects A_4_-GAPDH from irreversible oxidation and glutathionylated A_4_-GAPDH was reported to be efficiently reactivated by GRXs (Zaffagnini et al., 2008; Couturier et al., 2009; Gao et al., 2010). Similar results were reported for the cytoplasmic GAPDH isoform (GAPC) (Holtgrefe et al., 2008; Bedhomme et al., 2012). By contrast, the A_2_B_2_-GAPDH isoform and its higher oligomeric state A_8_B_8_-GAPDH were not found to undergo glutathionylation *in vitro* (Zaffagnini et al., 2007). Chlamydomonas TPI was also found to be glutathionylated *in vitro* but with no apparent effect on the enzyme activity (Zaffagnini et al., 2013a). Finally, glutathionylation is also likely affecting the CBC indirectly through regulation of TRXf (Michelet et al., 2005). Among all chloroplastic TRXs, TRXf from diverse species specifically undergo glutathionylation on a strictly conserved extra cysteine that is distinct from the two-active site cysteines and located in the vicinity of the active site. The glutathionylation of TRXf strongly decreases its ability to activate A_2_B_2_-GAPDH and NADP-MDH likely by perturbing the interaction with FTR since glutathionylated TRXf is less efficiently reduced in the light. This suggests that glutathionylation could affect all TRXf targets which include many enzymes involved in carbon fixation and other chloroplast metabolic pathways. A_2_B_2_-GAPDH being specifically activated by TRXf, GAPDH activity is likely fully down-regulated under conditions leading to protein glutathionylation in chloroplasts, such as enhanced ROS production either by direct glutathionylation of A_4_-GAPDH on its catalytic cysteine or indirectly through glutathionylation of TRXf that decrease the activation of A_2_B_2_-GAPDH.

All CBC enzymes were also identified as nitrosylated proteins by proteomic approaches (Table 1). None of these putative regulations has been confirmed biochemically with the exception of Chlamydomonas TPI that was shown to be partially inhibited by nitrosylation (Zaffagnini et al., 2013a) and land plant Rubisco which appears to be inhibited by nitrosylation (Abat et al., 2008; Abat and Deswal, 2009). Cytosolic GAPDH was shown to be completely inhibited by nitrosylation and fully reactivated by GSH or, less efficiently, by TRXs (Holtgrefe et al., 2008; Zaffagnini et al., 2013b). These properties may likely apply to chloroplastic GAPDH considering its strong structural and biochemical similarities with cytosolic GAPDH.

**Figure 8:**
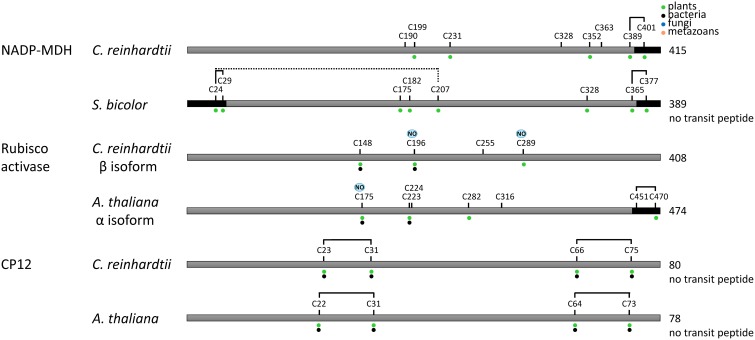
**Redox regulated cysteines in proteins controlling indirectly the Calvin–Benson cycle.** All proteins are schematically represented linearly. The numbering corresponds to the sequence with the transit peptide, except when “no transit peptide” is indicated on the right. Lines between cysteines indicate confirmed (black) or suggested (black and dashed) disulfide bonds. Cysteines identified as nitrosylated and glutathionylated are labeled with NO (blue) and SG (purple), respectively. The dots indicate cysteine conservation in photosynthetic organisms (green), bacteria (black), fungi (blue) and metazoans (orange). In *A. thaliana* Rubisco activase, the regulatory cysteines Cys451 and Cys470 in the precursor correspond to Cys392 and Cys411 in the mature form. Dark boxes correspond to insertions/extension present in the TRX-regulated isoforms and absent in the isoforms not regulated by TRX.

Recent proteomic studies also allowed identification of the cysteine residues undergoing nitrosylation and glutathionylation (Table 1). A schematic representation of the sites identified as nitrosylated, glutathionylated or forming a TRX-reducible disulfide bond is presented in Figures 7, 8. Many of these cysteines are conserved, especially in photosynthetic organisms. Some sites are shared between different modifications while others are unique. For example, in Chlamydomonas PRK, Cys47 appears modified by the 3 types of redox PTMs while Cys274 was only found to undergo glutathionylation. By contrast, Chlamydomonas FBPase is modified on 4 distinct cysteines that are all conserved in photosynthetic organisms: the enzyme undergoes glutathionylation on Cys109, nitrosylation on Cys233 while the TRX-reducible disulfide bond is most likely located between Cys213 and Cys219 by homology with higher plant FBPase. The large subunit of Rubisco appears to undergo multiple modifications with Cys459 identified as nitrosylated and 3 cysteines (Cys172, Cys247, Cys427) undergoing both nitrosylation and glutathionylation (Table 1, Figure 7). These results are consistent with previous studies (reviewed in (Moreno et al., 2008)) that suggested a redox control of the activity and/or the stability of Rubisco involving Cys172 (Moreno and Spreitzer, 1999; Marcus et al., 2003), Cys427 (Muthuramalingam et al., 2013) or Cys 459 (Marin-Navarro and Moreno, 2006). The multiplicity of the modification sites suggest that CBC enzymes are indeed regulated by multiple redox PTMs although the different modifications may not occur at the same time, at the same site, to the same extent or under the same physiological/cellular conditions.

CBC enzymes appear to be regulated by an intricate network of redox PTMs whose dynamics remains to be explored. These regulations may allow a tight coupling between the activity of CBC enzymes and the intracellular redox state linked to environmental conditions. While dithiol/disulfide exchange reactions controlled by TRXs allow light-dependent activation of CBC enzymes, glutathionylation could constitute an alternative mechanism of regulation of the CBC pathway occurring under illumination and dependent on ROS production and glutathione. Indeed, all available data suggest that glutathionylation down-regulates the activity of numerous CBC enzymes. Therefore, it has been proposed that glutathionylation could constitute a new mechanism of regulation of photosynthetic metabolism allowing a fine tuning of the CBC cycle in order to redistribute reducing power (in the form of NAPDH) and energy (in the form of ATP) within chloroplasts under oxidative stress, thereby favoring ROS scavenging (Michelet et al., 2005; Lemaire et al., 2007). This redistribution may be required transiently to cope with stress conditions. Glutathionylation also constitutes a mechanism of protection of CBC enzymes containing highly reactive cysteines from irreversible oxidation in the presence of ROS, as demonstrated for GAPDH (Zaffagnini et al., 2007). It has also been proposed that the glutathionylation/deglutathionylation cycle catalyzed by GRXs may contribute to ROS scavenging within chloroplasts (Zaffagnini et al., 2012b). In addition to regulation of CBC enzymes, redox signaling contributes to numerous short- and long-term acclimation responses that allow plants to adapt to fluctuating environmental conditions by enabling metabolic readjustments to maintain cellular homeostasis (Scheibe and Dietz, 2012).

It should be kept in mind that redox PTMs of CBC enzymes do not necessarily imply that the modification regulates the CBC pathway. First, it is possible that the modification does not affect the activity of the protein as shown for the glutathionylation of Chlamydomonas TPI (Zaffagnini et al., 2013a). However, even if it does, the extent of the modification *in vivo* being undetermined, it is possible that it only affects a minor pool of the total protein (especially for abundant proteins such as those involved in the CBC) and modification of this pool may not be limiting for the pathway. Finally, numerous identified proteins may represent moonlighting proteins that, upon redox PTMs, are diverted to new functions unrelated to their metabolic role in carbon metabolism as shown for cytosolic GAPDH in mammals (Hara et al., 2005) (see Zaffagnini et al. this series). Indeed, upon apoptotic stimulation, nitrosylation of mammalian GAPDH triggers its translocation to the nucleus where it regulates gene expression through several mechanisms including transnitrosylation of nuclear proteins (Kornberg et al., 2010). Plant cytosolic GAPDH was also shown to undergo nitrosylation and to relocalize to the nucleus under stress condition but the exact physiological function of the modification remains to be established (Holtgrefe et al., 2008; Vescovi et al., 2013; Zaffagnini et al., 2013b). Moreover, the glutathionylation of mammalian GAPDH has been shown to regulate endothelin-1 (ET-1) expression by altering the binding of GAPDH to the 3′ untranslated region of ET-1 mRNA thereby increasing its stability and resulting in increased ET-1 protein levels and endothelial vasoconstriction (Rodriguez-Pascual et al., 2008). The only CBC enzyme for which a moonlighting function has been reported is Rubisco. Indeed, under oxidative stress the Rubisco holoenzyme, composed of 8 small subunits (SSU) and 8 large subunits (LSU) disassembles into its constituents and LSU subsequently binds chloroplast mRNAs non-specifically and forms large particles (Yosef et al., 2004; Knopf and Shapira, 2005; Cohen et al., 2006). Recently, Chlamydomonas LSU but not SSU was shown to accumulate in chloroplast stress granules (cpSGs) under oxidative stress conditions (Uniacke and Zerges, 2008). cpSGs are RNA granules related to mammalian stress granules that form during oxidative stress and disassemble during recovery from stress. This study therefore suggested a novel function of Rubisco LSU as an mRNA-localizing and assembly factor of cpSGs (Uniacke and Zerges, 2008). This moonlighting function being triggered by oxidative stress conditions, it may likely be regulated by redox PTMs. Since cysteine modifications control several moonlighting functions of cytosolic GAPDH, the same may be true for chloroplastic GAPDH isoforms participating in the CBC which also undergo multiple redox PTMs. These redox PTMs are likely to play an important role in ROS sensing and to allow adaptation or alternatively trigger programmed cell death under varying environmental conditions.

## Concluding remarks

Recent proteomic studies suggest that CBC enzymes undergo multiple types of redox PTMs including nitrosylation, glutathionylation and oxido-reduction of disulfide bonds. The possible regulations by additional redox PTMs whose importance starts to emerge in non-photosynthetic organisms, such as sulfenylation, sulfhydration or cysteinylation, will also have to be explored. Many data also suggest the existence of a strong interplay between the different types of redox PTMs as recently described for nitrosylation and glutathionylation (Zaffagnini et al., 2012c). All these data suggest that CBC enzymes are regulated by a complex and highly dynamic network of redox PTMs. Unraveling the importance and function of these redox modifications under diverse physiological and growth conditions and characterizing the underlying molecular and structural determinants will certainly constitute a major challenge for future studies. Acquiring this knowledge is highly desirable considering the central role of the CBC in the determination of crop yields, CO_2_ fixation, biomass and biofuel production and plant adaptation to stress conditions.

### Conflict of interest statement

The authors declare that the research was conducted in the absence of any commercial or financial relationships that could be construed as a potential conflict of interest.
